# Supplementary material for: DeepRank: a deep learning framework for data mining 3D protein-protein interfaces
Source: Nat Commun. 2021 Dec 3;12:7068. doi: 10.1038/s41467-021-27396-0 (PMC8642403; doi:10.1038/s41467-021-27396-0)
Supplement: Supplementary file 1 — Supplementary Information [file 41467_2021_27396_MOESM1_ESM.pdf]

Supplementary Information for:

DeepRank:

A deep learning framework for data mining 3D protein-protein interfaces

Nicolas Renaud[1], Cunliang Geng[1,2], Sonja Georgievska[1], Francesco Ambrosetti[2], Lars Ridder[1], Dario F. Marzella[3], Manon F. Réau[2], Alexandre M.J.J. Bonvin[2] *, Li C Xue[2,3] *

1 Netherlands eScience Center, Science Park 140, 1098 XG, Amsterdam, The Netherlands

2 Bijvoet Centre for Biomolecular Research, Faculty of Science - Chemistry, Utrecht University, Padualaan 8, 3584 CH Utrecht, The Netherlands

3 Center for Molecular and Biomolecular Informatics, Radboudumc, Greet Grooteplein 26-28, 6525 GA Nijmegen, The Netherlands

*** Corresponding authors: Li C Xue (me.lixue@gmail.com); Alexandre Bonvin (a.m.j.j.bonvin@uu.nl)**

Table of Contents

Supplementary Note 1 2
Supplementary Note 2 4
Supplementary Note 3 6
Supplementary Note 4 14
Supplementary References 17

Supplementary Note 1

DeepRank is built as a comprehensive Python3 package that allows end-to-end classification and/or ranking of protein-protein interfaces. The package is accessible on GitHub: https://github.com/DeepRank/deeprank. DeepRank and its dependencies can easily be installed using the PyPI package manager using the command:

```
pip install deeprank
```

The global architecture of the software is represented in Supplementary Figure 1. The two main components of DeepRank are respectively in charge of the feature calculation and of the training of the models. These two components communicate via dedicated HDF5 files containing the mapped features that are used as input for the training. Detailed descriptions on how to use the code can be found in the online documentation: https://deeprank.readthedocs.io/

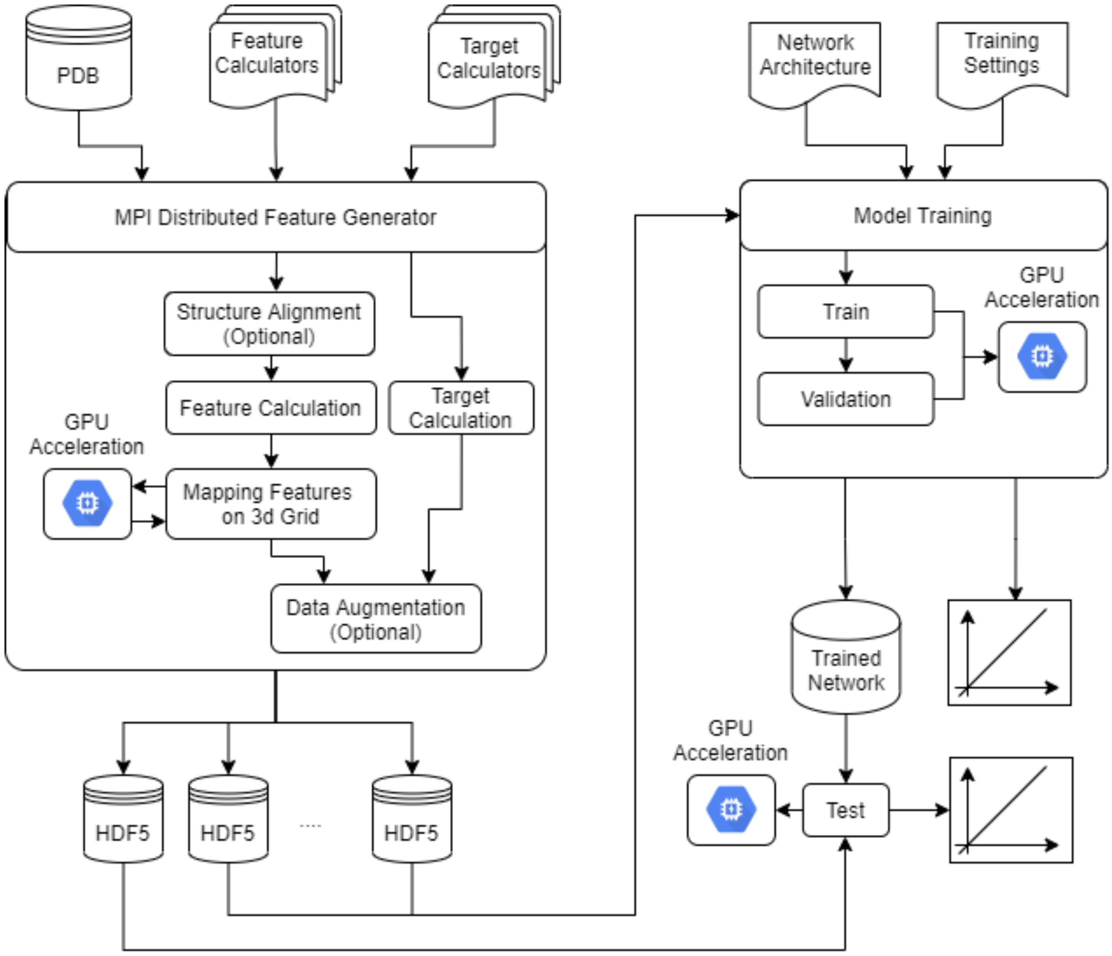

Supplementary Figure 1. Architecture of the DeepRank software. The two main blocks are respectively in charge of 1) 3D feature grid generation and 2) training and evaluating of the network. The package contains two main parts: one dedicated to the generation of 3D feature grids and the other dedicated to the training of neural networks.

Data Generation Module: The data generating module takes as inputs the PDB files of the complexes/models used for training/validation/testing. The features listed in Table 1 and targets (e.g., class labels in the context of classification, or binding affinities in the context of affinity predictions) presented in the main text are all implemented in DeepRank and can simply be called while instantiating the `DataGenerator` object. If users need new features and/or targets, these can be easily added to be used by the data generator. A tutorial explaining this process is available in the online documentation:

https://deeprank.readthedocs.io/en/latest/advTuto.html#

Once PDB files, features and targets metrics are specified, the data generator will split the processing of the PDB files among the requested number of MPI processes. This can greatly accelerate the data pre-processing and featurization process. Since it is essentially embarrassingly parallel it scales linearly with the number of MPI processes. For each PDB file, the feature values will be first calculated. These values are localized on a given atom or residue. They are subsequently mapped on a 3D Grid using a Gaussian mapping (see Methods in the main manuscript).

The mapping process is supported by MPI distributed processes and GPU offloading through dedicated CUDA kernels implemented in DeepRank to ensure efficient computations for very large datasets. As an indication of the processing time, mapping one feature on a 30x30x30 grid requires 0.135 s on a single CPU (Intel(R) Xeon(R) CPU E5-2650 v4 @ 2.20GHz) compared to 0.085 s on an NVIDIA GeForce Ti1080. A larger speed-up is expected for grids containing more points. Note that, in order to reduce the size of the HDF5 files, the 3D grids can also be computed on demand during the training of the model, for example to change the grid size and/or resolution. However, this comes at the price of increased computational costs for training a model. The target values are also calculated using either the native methods implemented in DeepRank or user-defined ones.

Data augmentation is supported in DeepRank by randomly rotating the 3D structure of the complex around the geometric center of its interface, after which features are automatically mapped onto the grid. Users can specify a given number of copies for each structure: Each copy will be randomly rotated to augment the data set and expose the network to different orientations of the same interface. For situations where random orientations of the PPIs are not desired, protein complexes can be aligned by DeepRank using Principal Component Analysis (PCA)-based alignments along cartesian axes.

All resulting feature and target data are stored in a series of HDF5 files. The data set is split into multiple HDF5 files, one file for each MPI process used. Hence, to compute the features of docking models of 1 given complex using 24 MPI process, 24 HDF5 files will be created each containing the features of a subset of docking conformations.

The HDF5 file format is increasingly popular for storing large datasets used in deep learning applications as it allows for efficient memory usage and fast input/output operations. In addition all the required metadata (code versioning, protocol generation and so on) can be directly included in the HDF5 file which partly aligns with the FAIR principle (Findable, Accessible, Interoperable and reusable)

Using HDF5 files also allows to easily explore the data sets using our in-house HDF5 browser DeepXplorer (https://github.com/DeepRank/DeepXplorer) specifically tuned for the data generated here. This graphical user interface allows to easily browse and visualize the mapped features through popular molecular viewers such as VMD and PyMol.

Training Module: The module in charge of the training of the model can easily read the data contained in the HDF5 files. It can specify which HDF5 file to use in training/validation and testing and can also select complexes based on specific criteria, such as, models with iRMSD larger and/or lower than a given threshold. The training module also needs as input a Python file containing the architecture of the neural network to be used. An example file is provided with the package (i.e., `deeprank/learn/model3d.py`, which contains simple 3D CNN architectures for classification and regression) but users can easily expand on this architecture to optimize the hyper-parameters of the model.

The data set resulting from the selection of a subset of conformations is then used to train the model. Note that the dataset is not loaded in memory. Instead, each data point is dynamically loaded when needed in the minibatch. At the end of the training, a plot of losses over epochs is generated assisting the user to identify the optimal network parameters. The best models with the lowest losses are stored together with the training data in a dedicated HDF5 file. This allows easy exploration of the training and validation performance through the HDF5 browser. The model be then used on an independent part of the total data set. For classification, DeepRank outputs boxplots of prediction scores and accuracy plots for train/validation/test. For regression, DeepRank produces scatter plots of predicted values against target values.

Supplementary Note 2

We provide here details about the architecture and training procedure used for the classification of biological interfaces vs. crystal ones.

Network Architecture: The network used for this task is summarized in Supplementary Figure 2. The network contains two 3D CNN/Max pooling blocks followed by two linear fully connected layers. This results in 117,362 learnable parameters. 80% of 5739 complexes from the MANY data set were used as training set. Each complex was augmented with 30 rotations leading to a total of 142352 conformations. The remaining 20% of the MANY data set, i.e., 1147 conformations were used in the validation data set. The test set was composed of the 161 conformations from an independent data set, namely the DC dataset. PSSM were used as features for residues in each chain (i.e., each residue is represented as 20 by 1 vector), leading to a total of 40 input channels.

```
Data Set Info:
Augmentation        : 30 rotations
Training set        : 142352 conformations
Validation set      : 1147 conformations
Test set            : 161 conformations
Number of channels  : 40
Grid Size           : 10, 10, 10
----------------------------------------------------------------
        Layer (type)               Output Shape         Param #
================================================================
            Conv3d-1          [-1, 80, 9, 9, 9]          25,680
         MaxPool3d-2          [-1, 80, 4, 4, 4]               0
            Conv3d-3         [-1, 120, 3, 3, 3]          76,920
         MaxPool3d-4         [-1, 120, 1, 1, 1]               0
            Linear-5                  [-1, 120]          14,520
            Linear-6                    [-1, 2]             242
================================================================
Total params: 117,362
Trainable params: 117,362
Non-trainable params: 0
```

Supplementary Figure 2. Architecture of the neural network and dataset size used for classification of biological vs. crystal interfaces.

Loss and early stopping: Supplementary Figure 3 shows the training and validation losses over epochs. As the validation loss is lowest at epoch 2, we used the trained network at epoch 2 as our final network. We then applied the trained network on an independent set, the DC set, achieving 86% accuracy (see Fig. 2).

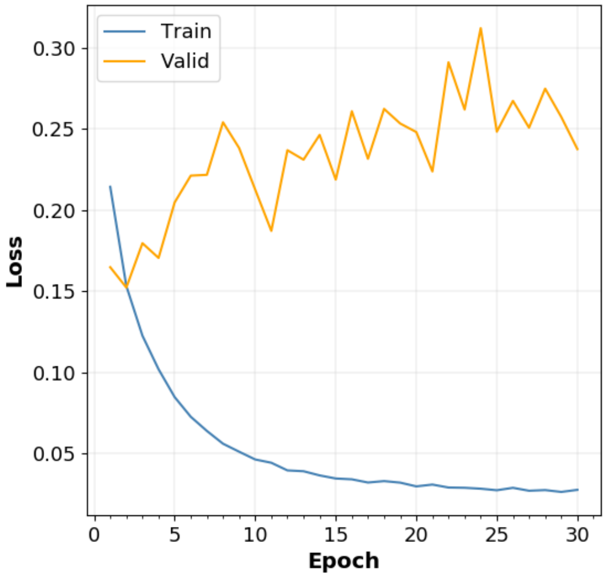

Supplementary Figure 3. Losses of training and validation on the MANY dataset for classification of biological vs crystal interfaces.

Redundancy of DC set and related DeepRank performance: To explore the redundancy of the DC test dataset, we have calculated sequence identities of DC complexes against those from the MANY dataset on chain level. We used 30%, a standard cutoff for sequence identity to define a homolog. As a result, 89 out of the 161 complexes from the DC set have at least 1 homolog in the MANY set. These 89 complexes have each on average 4 homologs in the training set. This consequently leaves 72 complexes from the DC set that do not have any homologs in the MANY set, the training set.

We have then evaluated the performance of the trained model on the 72 complexes of the DC dataset that do not present any homologs. From Supplementary Table 1, we can see that DeepRank reaches an accuracy of 81.9% even without homologs in the training data set. This therefore excludes the risk that the performance of our model is due to the presence of homologous sequences in the training and test data set.

Supplementary Table 1. Performance on redundant and non-redundant DC complexes.

	Accuracy	TP	FN	TN	FP	#complexes
DC complexes without homologs	81.9%	32	9	27	4	72
DC complexes with homologs	88.7%	34	5	45	5	89
All DC complexes	85.7%	66	14	72	9	161

Supplementary Note 3

In this note we present the details about the methods we used to train the neural network for the ranking problem as well as additional results.

Data Selection: As mentioned earlier, the MPI-supported feature grid generation produces a series of HDF5 files. We ran the data generation using 24 MPI processes leading to 24 HDF5 files for each complex. For example, the 25000 docking conformations of PDB 3AAD were split into 24 HDF5 files, named 000_3AAD, 001_3AAD, …. 024_3AAD, each file containing all the mapped features, targets, etc. for about 1000 different docking conformations. As the conformations were not clustered, a large degree of redundancy exists between the conformations stored in the different files. After experimentation we concluded that considering a subset of models consisting of 3 HDF5 files per complex was sufficient to capture the distribution of the whole dataset (001, 002 and 003, ~0.4 million models). Supplementary Figure 4 shows the distribution of iRMSD values of the whole dataset (gray shaded area) and of the portion used during our experiments. The left panel shows the distribution aggregated for all complexes while the right panel shows individual cases.

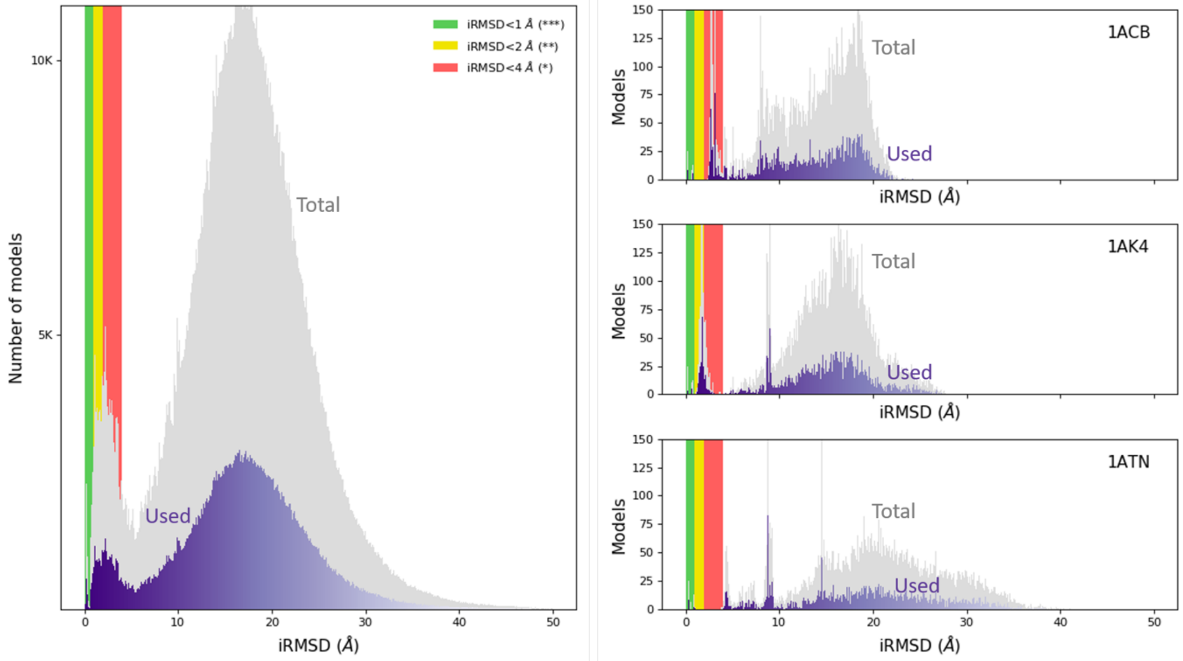

Supplementary Figure 4. Distribution of the iRMSD values. The gray shaded area shows the distribution for the whole data set, while the purple area displays the distribution of the limited portion used during our experiments. The selected data reliably represents not only the entire dataset (left panel) but also individual cases (right panel). The histograms of the total dataset have been scaled by a factor ½ for clarity.

Neurak Network Architecture: The network used for the ranking problem is summarized in Supplementary Figure 5. The network was composed for 8 sequential layers: a succession of 3D CNN, max-pooling and 3D batch normalization with 2 fully connected layers. It contains 7232 trainable parameters.

```
    Data Set Info:
    Training set               : 338389 conformations
    Augmentation               : 0 rotations
    Validation set             : 40410 conformations
    Test set                   : 39425 conformations
    Number of channels         : 36
    Grid Size                  : 30, 30, 30
----------------------------------------------------------------
        Layer (type)               Output Shape         Param #
================================================================
       BatchNorm3d-1       [-1, 36, 30, 30, 30]              72
            Conv3d-2        [-1, 6, 28, 28, 28]           5,838
       BatchNorm3d-3        [-1, 6, 28, 28, 28]              12
         MaxPool3d-4           [-1, 6, 9, 9, 9]               0
            Conv3d-5           [-1, 6, 7, 7, 7]             978
       BatchNorm3d-6           [-1, 6, 7, 7, 7]              12
         MaxPool3d-7           [-1, 6, 2, 2, 2]               0
            Linear-8                    [-1, 6]             294
       BatchNorm1d-9                    [-1, 6]              12
           Linear-10                    [-1, 2]              14
================================================================
Total params: 7,232
Trainable params: 7,232
Non-trainable params: 0
```

Supplementary Figure 5: Architecture of the neural network and dataset size for the docking scoring problem.

The performances reported in Fig. 3 (and Supplementary Figure 6) were achieved in one epoch on 10-fold cross validation. For each epoch the training took 2 hours on a 16-core CPU and 1 GPU cards. We used ~340K models for training, 40K for validation and 40K for testing. We used the full set of 36 physico-chemical features (channels) that are predefined in DeepRank (Table 1).

Performance comparison between HADDOCK and DeepRank on BM5: Supplementary Figure 6 shows the Hit Rate obtained with DeepRank and HADDOCK Score on models generated in the rigid-body docking stage only using the HADDOCK-it0 scoring function (left panel) and HADDOCK-itw scoring function (right panel). Using HADDOCK-it0 scoring function on models generated in the rigid-body docking phase leads to similar performance than DeepRank. However, using HADDOCK-itw scoring function on the same models, i.e. rigid-body docking models, leads to significantly lower performance. This illustrates the robustness of DeepRank on different model qualities.

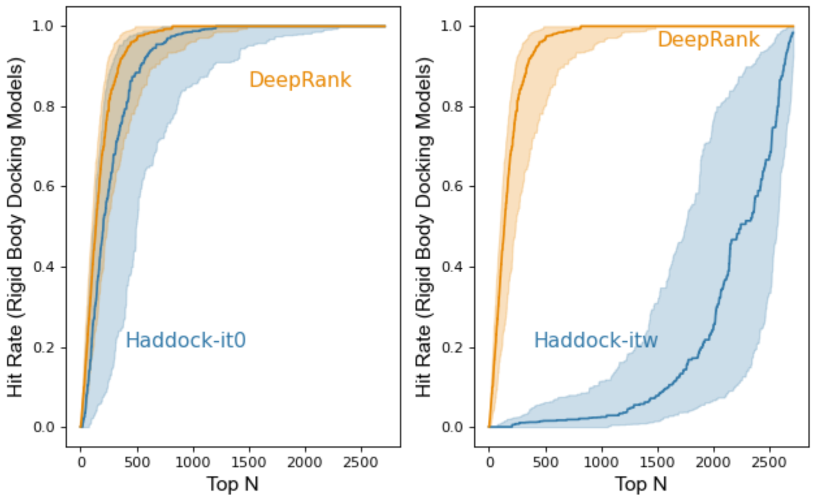

Supplementary Figure 6. Comparing DeepRank and different HADDOCK scoring functions on rigid-body docking models. DeepRank and HADDOCK-it0 show similar performance on these models (left). However HADDOCK-itw does not perform well on rigid-body-docking models (right). The shaded area marks the 25% - 75% quantile interval.

We further checked the performance of DeepRank and HADDOCK Score on models originating from different docking stages: rigid-body docking, semi-flexible docking, and water refinement (Supplementary Figure 7). We used the most suitable HADDOCK scoring function in each case i.e. it0 for rigid-body docking models, it1 for semi-flexible docking models and itw for water refined models. DeepRank and HADDOCK show comparable performance with a slight advantage for DeepRank in each case when considering an increasing number of topN models.

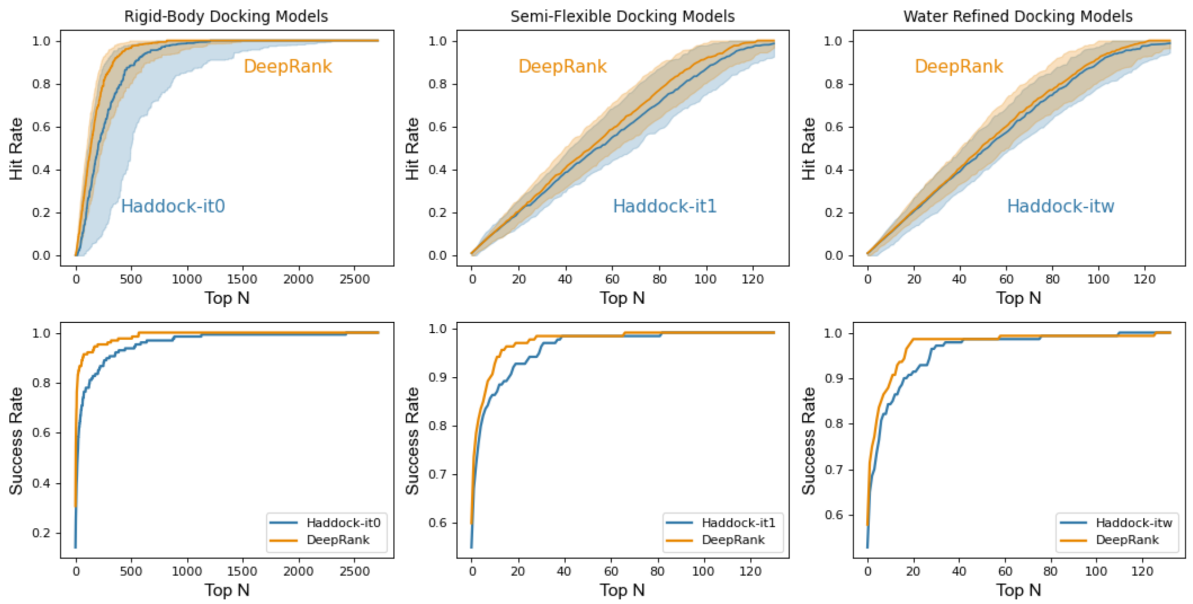

Supplementary Figure 7. Ranking performance of DeepRank and HADDOCK. Hit Rate over complex (top row) and Success Rate (bottom row), on models generated in different docking stages; rigid-body docking models (left); semi-flexible docking models (middle); water-refined models (right). The shaded area in the Hit Rate plots marks the 25% - 75% quantile interval.

Performance comparison on CAPRI score set: To further test the performance of DeepRank we have trained a final 3D CNN model using the conformations of all the 142 BM5 complexes and applied it to 13 cases from the CAPRI score set[1]. The CAPRI score set was generated by various docking software and represents an independent test set. We compared the DeepRank results to three leading scoring functions, the HADDOCK scoring function, the recently developed iScore[2], a graph-kernel based scoring function, and DOVE, a deep learning framework that also uses 3D CNN but different features[3]. We have used here the so-called ATOM20 score given by DOVE as it seems to be the most competitive in our calculations in agreement with the results reported in the original paper. To ensure optimal performance of the HADDOCK scoring function, the models of the CAPRI data set were subjected to a short energy minimization to remove clashes produced by rigid-body docking methods.

DeepRank is generally competitive with HADDOCK, iScore and DOVE (Supplementary Figure 8), performing better than the other methods on some cases especially in the top 200 and beyond (Supplementary Table 2). DeepRank also performs very well when only a limited number of near-native models are present in the data set as is the case for T30 and T35 (Supplementary Figure 8) (2 out of 1343 for T30 and 3 out of 499 for T35, respectively). This suggests the ability of DeepRank to correctly identify favorable interactions that are ignored by the other methods and indicates a possible complementarity of these approaches.

The complementarity of DeepRank to other scoring approaches is further illustrated by comparing the rankings given by DeepRank and HADDOCK per cases as represented in Supplementary Figure 9. The lower a ranking the more likely HADDOCK/DeepRank considers

a model to be near-native. As seen in these figures, the rankings given by DeepRank and HADDOCK can be significantly different. In cases of T40, T47, T50 and T53, a cluster of near-native conformations appears on the top left corner. This shows that DeepRank correctly ranks these conformations while HADDOCK mistakenly predicts them as wrong models, mainly because of clashes resulting in high van der Waals energies. As an example, conformations of T50 are illustrated in Supplementary Figure 10. We can see that these conformations only differ by variations of the orientation of one chain with respect to the other one. This illustrates the large variability of the different scoring functions with respect to structural changes.

The success rate reported in the last row of Supplementary Table 2 show that the different methods perform overall similarly. Only small differences appear: iScore performs the best in the Top10 with a success rate of 54% but DeepRank outperforms the other methods in the Top200 with a success rate of 92%.

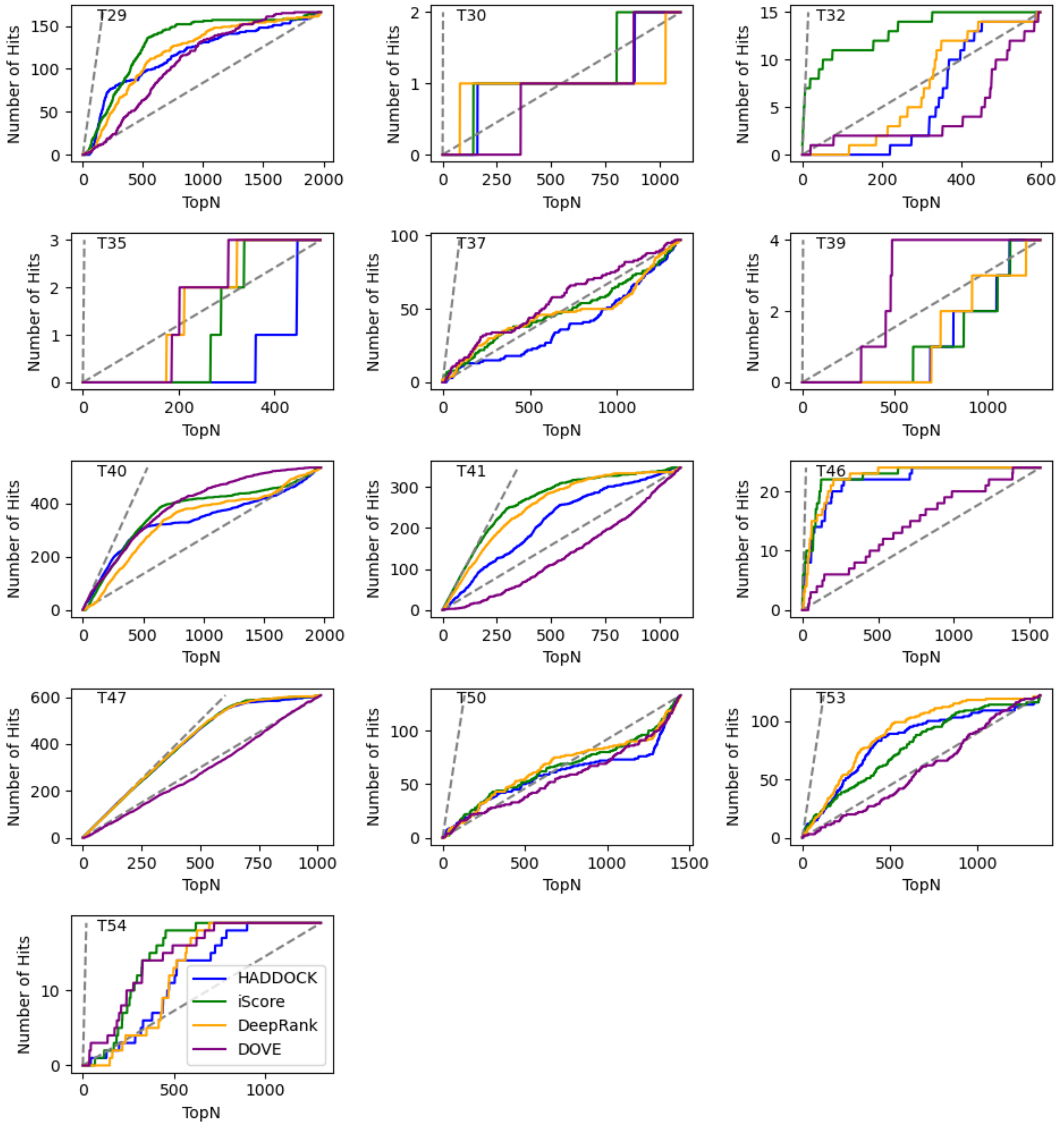

Supplementary Figure 8. Hit Rate plot of the 13 CAPRI cases given by HADDOCK, iScore, DOVE and DeepRank. DeepRank is competitive with these three scoring functions.

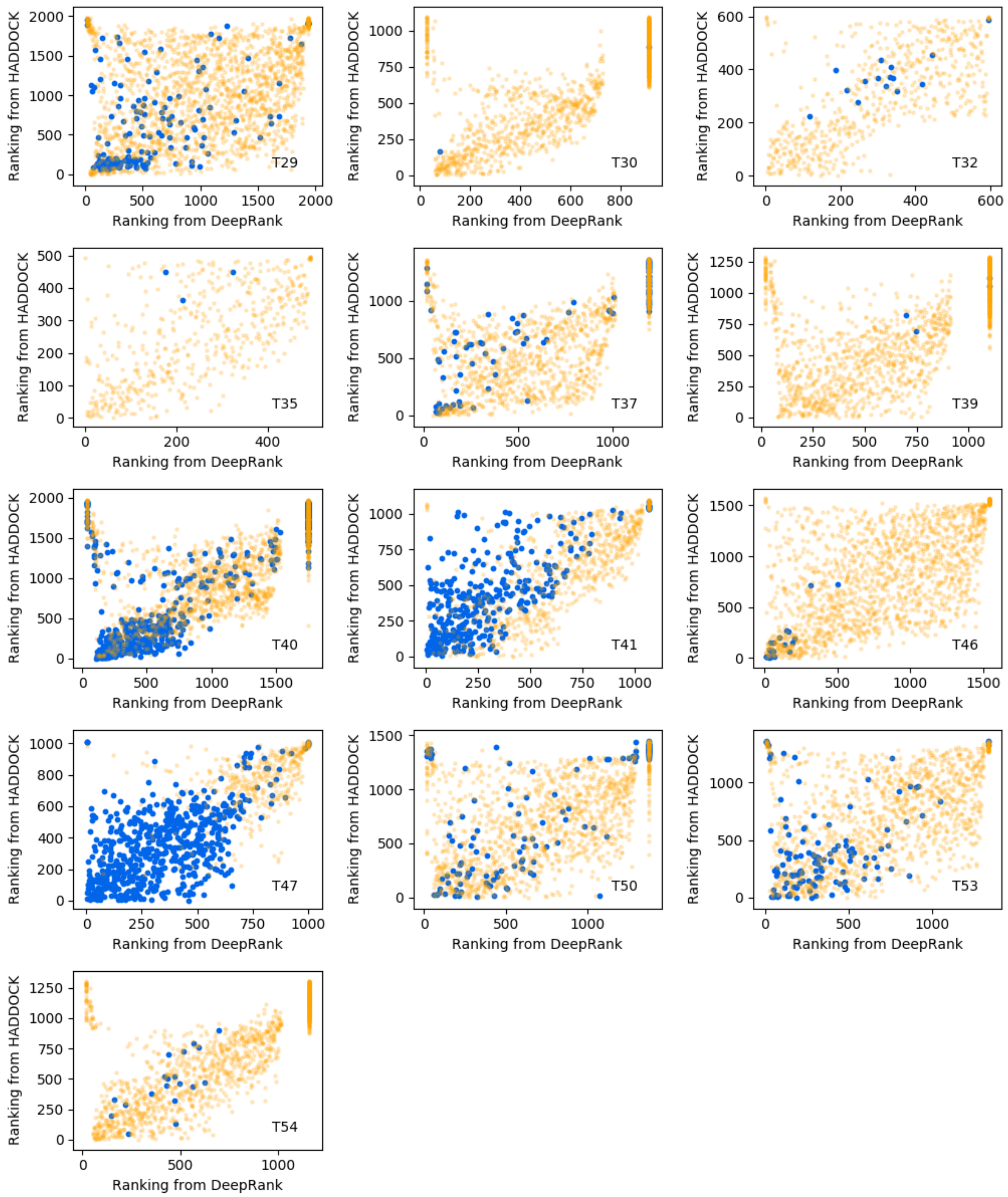

Supplementary Figure 9. Comparison of the rankings obtained with HADDOCK and DeepRank on the CAPRI score set. The lower a ranking the more likely HADDOCK/DeepRank considers a model to be near-native. Blue points represent near-native models while orange ones mark wrong models. The cluster of blue dots in the top-left corner is near-native models correctly identified by DeepRank but missed by HADDOCK. Inversely, the blue dots in the lower-right corner are near-native models that are correctly identified only by HADDOCK. Additionally, clusters of near-native models in the top right corner are those that are misclassified as wrong models by both DeepRank and HADDOCK.

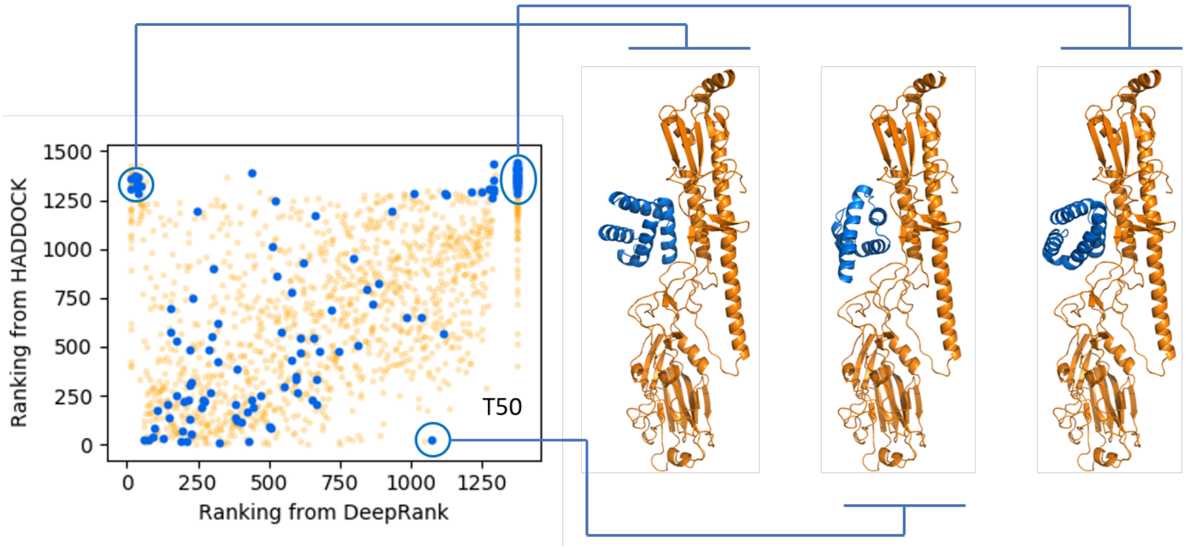

Supplementary Figure 10. Comparison of the rankings given by HADDOCK and DeepRank for the conformations of the T50 case. The blue/orange dots mark near-native/wrong models. This scatter plot illustrates near-native conformations that are correctly or incorrectly ranked by either or both of the two scoring functions.

Supplementary Table 2. Performances on 13 cases of the CAPRI score set. The number of near-native models among Top10, Top25 and Top200 are reported, showing the good performance of our trained 3D CNN in this task. On most cases the three methods perform similarly with only a few cases where the values differ significantly between the three approaches. Note that the models are not clustered, thus we evaluate a high range up to top 200 models. The number in parenthesis in the model column indicates the number of near native conformations. The bottom row (SR) indicates the overall success rate.

	Models	HADDOCK			iScore			DOVE			DeepRank		
	Total (native)	T10	T25	T200	T10	T25	T200	T10	T25	T200	T10	T25	T200
**T29**	1979 (166)	0	0	71	0	0	49	0	2	18	0	2	37
**T30**	1148 (2)	0	0	1	0	0	1	0	0	0	0	0	1
**T32**	599 (15)	0	0	0	7	8	12	0	1	2	0	0	2
**T35**	497 (3)	0	0	0	0	0	0	0	0	0	0	0	1
**T37**	1364 (97)	0	1	13	4	7	20	0	3	27	2	3	25
**T39**	1295 (4)	0	0	0	0	0	0	0	0	0	0	0	0
**T40**	1987 (535)	10	24	155	0	1	138	10	24	138	1	5	90
**T41**	1101 (347)	1	7	105	10	25	187	2	2	24	5	18	155
**T46**	1570 (24)	3	8	20	6	9	22	0	0	6	1	4	21
**T47**	1015 (608)	10	25	197	10	24	194	1	7	107	9	24	195
**T50**	1447 (133)	1	6	23	2	2	28	1	3	22	0	2	24
**T53**	1360 (122)	5	10	39	6	9	35	2	2	14	1	5	45
**T54**	1304 (19)	0	0	3	0	0	5	0	0	7	0	0	2
**SR**		46%	54%	77%	54%	61%	85%	39%	61%	77%	46%	61%	92%

Computational Efficiency on Data Generation: As mentioned in the main text, the data generation process is rather efficient within DeepRank. As an example, we compare in Supplementary Table 3 the time required to process PDB files in DeepRank and in MaSIF. As seen in this table DeepRank only requires a few seconds per complex while MaSIF can go up to about 12 minutes; DeepRank needs about 120MB memory on average while MaSIF requires more than 830MB. The experiments are done on AMD EPYC 7451 24-Core Processor. One CPU was used. Note that MASIF uses two geometric and three chemical features. Deeprank uses 36 physico-chemical features, out of which PSSMs (accounting for 20 features) were precalculated and provided as input.

Supplementary Table 3. Benchmark of data preprocessing time with DeepRank and MASIF on 123 protein complexes from BM5.

	**Data Preprocessing Speed** [second/complex]		**Memory** [megabyte]	
	**DeepRank**	**MaSIF**	**DeepRank**	**MaSIF**
min	3	8	117	91
max	15	722	133	2366
median	6	274	119	775
mean	6	288	119	835
std	2	129	2	400

Supplementary Note 4

To illustrate the usage and flexibility of DeepRank we present here a small tutorial explaining the two main stages of DeepRank, i.e., the 3D feature grid generation and the model training. A more extended version of this tutorial can be found on the online documentation on GitHub: https://deeprank.readthedocs.io/.

The snippet of code in Supplementary Figure 11 shows an example for the 3D feature grid generation through the `DataGenerator` class (line 15) that orchestrates the entire process. During the initialization of the class, users must provide: the path of the directory containing the PDB files (the `pdb_source` option) and the target values for each 3D model/structure (`add_unique_target()`). Users also need to provide the path to the directory containing the precalculated PSSM files (line 16; e.g., using https://github.com/DeepRank/PSSMGen) if they want to use PSSM as features. DeepRank has predefined a set of features, such as van de Waals. Users can also specify which features to be computed (line 18-22). Users may also define their target function(s) and let DeepRank to calculate a variety of targets (e.g., FNAT,

iRMSD) for each 3D structure/model. Several methods are readily implemented in DeepRank but it is possible to define new features and target, and include them in the database generation.

Once the database is initialized, calling the method `create_database()` (line 28) will generate a HDF5 file containing one group of PDB files where all its the relevant information (conformation, feature values target values) is stored. However, at this stage the features are not mapped onto the 3D grid. The mapping is performed subsequently by calling the method `map_features()` (line 37)

```
1  """
2  Generate 3D feature grids dataset in HDF5 format.
3  """
4
5  import os
6  from deeprank.generate import *
7  from mpi4py import MPI
8
9  def generate_dataset(pdb_source, pssm_source, bin_class, h5out):
10
11     if os.path.isfile(h5out):
12         os.remove(h5out)
13
14     # initialize the database
15     database = DataGenerator(pdb_source=pdb_source,
16                              pssm_source=pssm_source,
17                              #  compute_targets  = ['deeprank.targets.binary_class'], # users may define their own target functions
18                              compute_features = ['deeprank.features.AtomicFeature',
19                                                  'deeprank.features.FullPSSM',
20                                                  'deeprank.features.PSSM_IC',
21                                                  'deeprank.features.BSA',
22                                                  'deeprank.features.ResidueDensity'],
23                              data_augmentation = 30, # rotate complexes
24                              mpi_comm=MPI.COMM_WORLD,
25                              hdf5=h5out)
26
27     #create the database
28     database.create_database(prog_bar = True)
29
30     # define the grid parameters
31     grid_info = {
32        'number_of_points': [10, 10, 10],
33        'resolution': [3., 3., 3.],
34        'atomic_densities': {'C': 1.7, 'N': 1.55, 'O': 1.52, 'S': 1.8},
35     }
36     # map the features to 3D grid
37     database.map_features(grid_info, try_sparse=True, time=False, prog_bar=True)
38
39     # add target value
40     database.add_unique_target({'BIN_CLASS':bin_class})
41
42 if __name__ == '__main__':
43
44     # generate 3D feature grids for the positive dataset
45     generate_dataset('path/to/pdb_positive_class', 'path/to/pssm', 1 , 'path/to/output_positive.hdf5')
46     # generate 3D feature grids for the negative dataset
47     generate_dataset('path/to/pdb_negative_class', 'path/to/pssm', 0 , 'path/to/output_negative.hdf5')
```

Supplementary Figure 11: Snippet of code illustrating the generation of 3D feature grid database.

Once the features have been mapped and the database is complete, users can use their data (i.e., 3D feature grids) to train models. Relying on PyTorch, users have full flexibility to define their network architecture (Supplementary Figure 13), optimizer and loss function (Supplementary Figure 12). The snippet of code in Supplementary Figure 12 shows how to train a model using DeepRank.

After importing the DeepRank tools and the user-defined network, users can define the dataset they want to use during the training. To this end, the HDF5 file(s) must be provided to initialize a `DataSet` instance (line 14). During the initialization users can also specify which features (lime 19) and which target (line 20) to use during the training. It is therefore very simple to use only specific features or to switch between different targets when training models. It is also

possible to use only PPIs whose target values satisfy some conditions through the `dict_filter` option of `DataSet`.

This dataset, together with the architecture of the network model here called `mycnn`, can then be used to instantiate a `NeuralNet` class (line 29) that orchestrates the training of the model. The user must also specify the type of task to perform (line 32), i.e., regression or classification. By default, an MSE and cross entropy loss are used for regression and classification, respectively. It is possible to change the loss function to any loss supported by PyTorch (line 42). Similarly, a stochastic gradient descent optimizer is set by default but users are free to change that to any optimizer supported by PyTorch (line 43). Once all is set the call to the `train()` method (line 46) will train the model and compute training and validation loss. All the results will be stored in a dedicated HDF5 file for post processing.

```
1 """
2 Train, validate and test
3 """
4 import torch.nn as nn
5 import torch.optim as optim
6 from deeprank.learn import DataSet, NeuralNet
7 from my_network import mycnn
8
9 trainset = ['path/to/train_positive.hdf5', 'path/to/train_negative.hdf5']
10 validset = ['path/to/validation_positive.hdf5', 'path/to/validation_negative.hdf5']
11 testset = ['path/to/test_positive.hdf5', 'path/to/test_negative.hdf5']
12
13 # declare the dataset instance
14 data_set = DataSet(train_database=trainset,
15                    valid_database=validset,
16                    test_database=testset,
17                    mapfly=False,  # Features have been already mapped to grids
18                    # select_feature={'Feature_ind': ['AtomicFeature', 'BSA', 'ResidueDensity']},
19                    select_feature={'Feature_ind' : ['PSSM_*'] }, # select_feature='all': selecting all precalculated features
20                    select_target='BIN_CLASS',
21                    #dict_filter = {'IRMSD':'<5. or >10.'}, # select a subset of models as training set, for example, base on 'IRMSD' in the docking scenario.
22                    normalize_features=False,
23                    normalize_targets=False,
24                    pair_chain_feature=None,
25                    clip_features=False,
26                    tqdm=True) #tqdm: show progress bar
27
28 # create the network
29 model = NeuralNet(data_set=data_set,
30                   model=mycnn,
31                   model_type='3d',
32                   task='class', # 'class' for classification tasks, 'reg' for regression
33                   pretrained_model=None,
34                   cuda=True,
35                   ngpu=2,
36                   plot=True,
37                   save_hitrate=False, #this option is True for the docking scoring problem
38                   save_classmetrics=True,
39                   outdir='path/to/out_dir')
40
41 # optionally set the loss and the optimizer
42 model.criterion = nn.CrossEntropyLoss(weight = tensor([0.8000, 0.2000]), reduction='mean')
43 model.optimizer = optim.Adam(params = model.net.parameters())
44
45 # start the training
46 model.train(nepoch=30,
47             preshuffle=True,
48             preshuffle_seed=2019,
49             divide_trainset=None,
50             #divide_trainset = [0.8, 0.2, 0.0],
51             train_batch_size=32,
52             num_workers=10,
53             save_model='all', # or 'best' for the best model based on the validation set
54             save_epoch='all',
55             hdf5='path/to/output.hdf5'
56             )
```

Supplementary Figure 12: Snippet of code illustrating the model training.

As mentioned above, the architecture of the network is specified in a separate python file so that users can have full control on the architecture they use. An example of such file is shown in Supplementary Figure 13. This file contains the definition of a network class that is typical in PyTorch. One constraint is that the definition of this class must use an argument `input_shape` that specifies the shape of the input tensor. This enables the use of this network for different sets of features that are selected in the `DataSet` instantiation step. The network below is composed of a series of 3D convolution followed by a fully connected layer.

We use here a small function `_get_conv_output`, which determines the size of this fully connected layer. Note that a dedicated model generator (`modelGenerator.py`) can assist in the creation of the files describing the neural network architecture.

```
class mycnn(nn.Module):

    def __init__(self, input_shape):
        super(mycnn, self).__init__()

        self.conv1   = nn.Conv3d(input_shape[0], 4, kernel_size=2)
        self.maxpool = nn.MaxPool3d((2, 2, 2))
        self.conv2   = nn.Conv3d(4, 5, kernel_size=2)
        self.fc = nn.Linear(self._get_conv_output(input_shape), 1)

    def forward(self, x):
        x = self._forward_features(x)
        x = x.view(x.size(0), -1)
        return self.fc(x)

    def _forward_features(self, x):
        x = F.relu(self.conv1(x))
        return = self.conv2(x)

    def _get_conv_output(self, shape):
        inp = Variable(torch.rand(1, *shape))
        out = self._forward_features(inp)
        return out.data.view(1, -1).size(1)
```

Supplementary Figure 13: Snippet of code illustrating the definition of the neural network architecture.

Supplementary References

1. Lensink, M. F. & Wodak, S. J. Docking and scoring protein interactions: CAPRI 2009. *Proteins Struct. Funct. Bioinforma.* **78**, 3073–3084 (2010).
2. Geng, C. *et al.* iScore: a novel graph kernel-based function for scoring protein–protein docking models. *Bioinformatics* **36**, 112–121 (2020).
3. Wang, X., Terashi, G., Christoffer, C. W., Zhu, M. & Kihara, D. Protein docking model evaluation by 3D deep convolutional neural networks. *Bioinformatics* **36**, 2113–2118 (2020).
